# Future of cardiac diffusion magnetic resonance imaging: a bibliometric analysis

**DOI:** 10.3389/fmedt.2026.1801878

**Published:** 2026-10-13

**Authors:** Xinyi Wu, Yuelong Yang, Yongzhou Xu, Zhigang Wu, Hui Liu, Rui Chen

**Affiliations:** 1 Department of Radiology, The Sixth Affiliated Hospital, Sun Yat-sen University, Guangzhou, Guangdong, China; 2 Biomedical Innovation Center, The Sixth Affiliated Hospital, Sun Yat-sen University, Guangzhou, Guangdong, China; 3 Department of Radiology, Guangdong Provincial People's Hospital, Guangdong Academy of Medical Sciences, Southern Medical University, Guangzhou, Guangdong, China; 4 Guangdong Provincial Key Laboratory of Artificial Intelligence in Medical Image Analysis and Application, Guangzhou, Guangdong, China; 5 MSC Clinical & Technical Solutions, Philips Healthcare, Guangzhou, Guangdong, China; 6 Siemens Shenzhen Magnetic Resonance Ltd, Shenzhen, Guangdong, China

**Keywords:** bibliometric analysis, cardiac diffusion magnetic resonance imaging, clinical application, preclinical application, technical development

## Abstract

**Background:**

Cardiac diffusion magnetic resonance imaging (cDMRI) has become a valuable noninvasive tool for assessing cardiac microstructure.

**Objective:**

To perform a systematic and comprehensive bibliometric analysis of cDMRI to explore current status, research hotspots and potential future directions.

**Material and methods:**

A systematic search of Web of Science Core Collection (WoSCC) and PubMed was conducted to identify articles on cDMRI published between 2005 and 2024. All retrieved records were independently screened by two reviewers to exclude the articles unrelated to the topic about cDMRI. Co-citation, collaboration networks, burst keywords, and reference analyses were performed.

**Results:**

There were 359 articles searched in WoSCC and 88 articles in PubMed. Finally, 367 articles met the search criteria in both databases. The cardiac “diffusion tensor imaging” (cDTI) emerged as the most prominent keyword, with the highest occurrence count (191.0) and strongest citation bursts (6.8). The study by Nielles-Vallespin et al. showed the strongest citation burst (26.17), accumulating 163 total citations and an average of 20.38 citations per year, highlighting its substantial influence on the development of cDMRI research. The top-cited articles with the strongest citation bursts concentrated on the clinical application of cDTI, its sequence design, and cardiac electrophysiology based on cDTI. The trend topic analysis further revealed “deep learning” as an emerging research focus in cDMRI since 2022.

**Conclusions:**

To our knowledge, this is the first dedicated bibliometric analysis focusing on cDMRI. By systematically analyzing the current status, research hotspots, and future trends of cDMRI, it provided valuable insights to guide future investigations in this field.

## 1 Introduction

Cardiac diffusion magnetic resonance imaging (cDMRI) has emerged as a promising noninvasive tool for assessing *in-vivo* myocardial microstructure (1–3). Its derived parameters characterize water diffusion and myocardial tissue organization, reflecting the alignment of cardiomyocytes and sheetlets. For example, apparent diffusion coefficient (ADC) maps derived from cardiac diffusion-weighted imaging (cDWI) have demonstrated reliable performance in detecting acute myocardial edema (4, 5). cDWI can also detect myocardial fibrosis and assess its extents in various heart diseases without contrast agents (6–8). Cardiac diffusion tensor imaging (cDTI) has been used to evaluate myocardial microstructural dynamics, providing insight into cardiomyocyte organization (9–12). More recently, cDTI models have been applied to investigate cardiac electromechanics (13), analyzing cardiomyocyte strain or myofiber strain as a potential method for early detection of cardiac dysfunction (14, 15). Moreover, cDTI has been employed to simulate cardiac electrical activation, representing a novel direction in cDMRI research (16, 17). Additionally, cDMRI has enabled the measurement of cardiomyocyte size, representing a novel capability in cardiovascular MRI (18, 19). Together, these advancements demonstrate the clinical utility and promise of cDMRI.

Bibliometrics employs both qualitative and quantitative methods to analyze large scientific datasets, effectively identifying research hotspots and trends (20, 21). In contrast to conventional narrative reviews, bibliometrics provides a more systematic and objective analysis of research topics. This study utilizes bibliometric methods to comprehensively analyze research on cDMRI, sourced from the Web of Science Core Collection (WoSCC) in the past 20 years. The analysis encompasses original research and reviews across multiple domains, including clinical applications, MRI methodology, medical image analysis, and preclinical technologies.

The present study aimed to conduct a systematic and comprehensive bibliometric analysis of cDMRI to identify current status, research hotspots and potential future directions.

## 2 Materials and methods

### 2.1 Data collection and search strategy

The workflow of search strategy and screening was illustrated in Figure 1.

**Figure 1:**
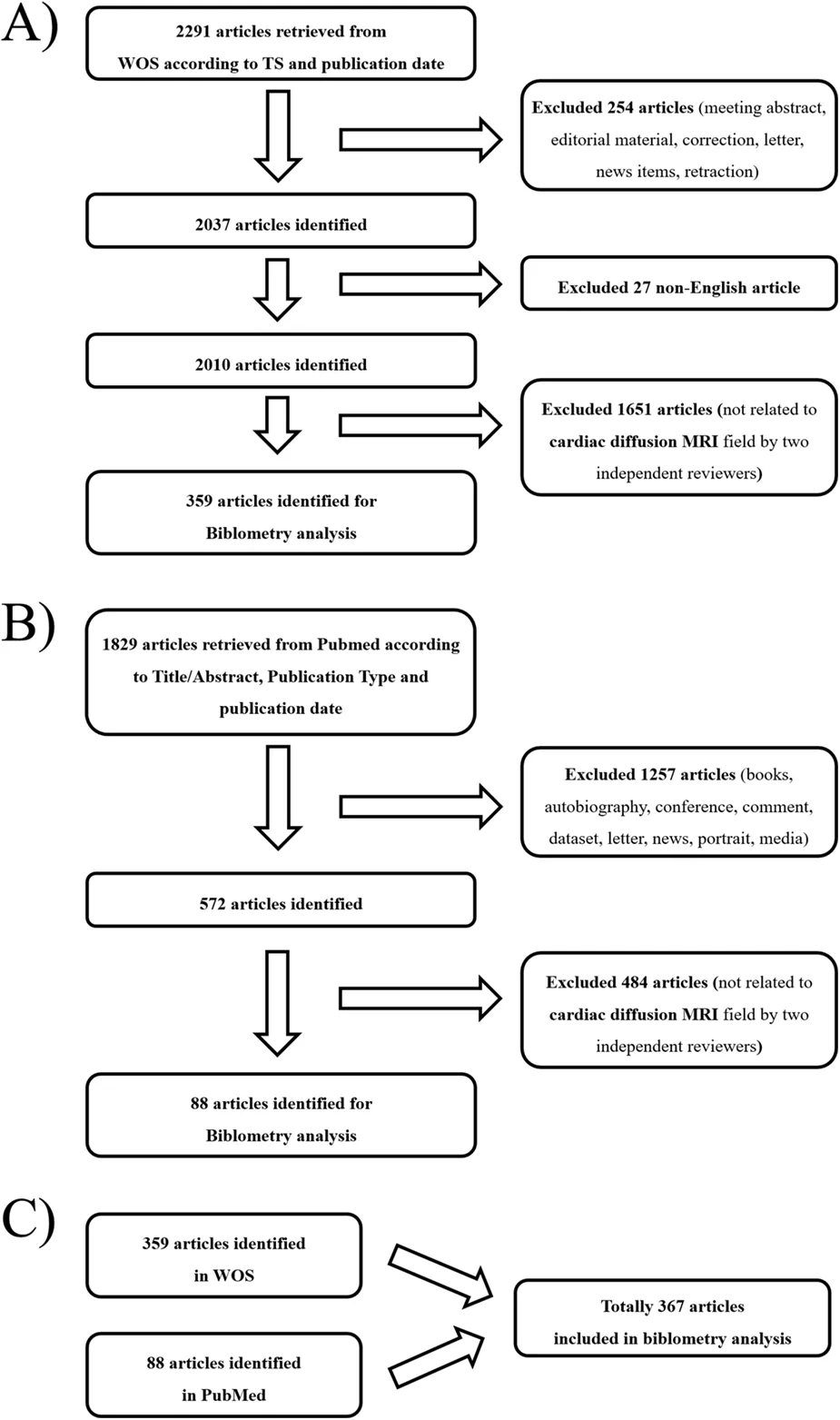
Flow diagram of article searching and screening progress using Web of science core collection (WoSCC) and pubMed databases. This study utilized bibliometric methods to comprehensively analyze research on Cardiac diffusion magnetic resonance imaging, sourced from the Web of Science Core Collection (WoSCC) and PubMed databases between 2005 and 2024. Articles included in the bibliometric analysis were original research and reviews published in English between January 1, 2005, and June 30, 2024. We did separate research from WoSCC and PubMed. All downloaded data were independently screened by reviewers. **(A)** Flow diagram of article searching and screening progress using WoSCC; **(B)** Flow diagram of article searching and screening progress using PubMed; **(C)** Results of article searching and screening using multiple databases.

#### 2.2.1 Search strategy

To retrieve a broader range of articles and minimize database-coverage bias, the articles from WoSCC and PubMed were searched in the present study. Articles included in the bibliometric analysis were original research and reviews published in English between January 1, 2005, and June 30, 2024. We did separate research from WoSCC and PubMed. The search of WoSCC and PubMed was described as follows.

The search of WoSCC was as follows: TS = (“cardiac” AND “diffusion tensor”) OR TS = (“heart*” AND “diffusion tensor”) OR TS = (“cardio*” AND “diffusion tensor”) OR TS = (“cardiac” AND “diffusion-tensor”) OR TS = (“heart*” AND “diffusion-tensor”) OR TS = (“cardio*” AND “diffusion-tensor”) OR TS = (“cardiac” AND “diffusion weight*”) OR TS = (“heart*” AND “diffusion weight*”) OR TS = (“cardio*” AND “diffusion weight*”) OR TS = (“cardiac” AND “diffusion-weight*”) OR TS = (“heart*” AND “diffusion-weight*”) OR TS = (“cardio*” AND “diffusion-weight*”) OR TS = (“cardiac” AND “intravoxel incoherent motion”) OR TS = (“heart*” AND “intravoxel incoherent motion”) OR TS = (“myocard*” AND “diffusion tensor”) OR TS = (“myocard*” AND “diffusion-tensor”) OR TS = (“myocard*” AND “diffusion weight*”) OR TS = (“myocard*” AND “diffusion-weight*”) OR TS = (“myocard*” AND “intravoxel incoherent motion”) AND LA = (English) AND (DT = “Article” OR DT = “Review”) AND Publication time span = (1 January 2005 to 30 June 2024).

The search of PubMed was as follows: (((“cardiac”[Title/Abstract] AND “diffusion-tensor”[Title/Abstract]) OR (“heart*”[Title/Abstract] AND “diffusion-tensor”[Title/Abstract]) OR (“cardio*”[Title/Abstract] AND “diffusion-tensor”[Title/Abstract]) OR (“cardiac”[Title/Abstract] AND “diffusion-tensor”[Title/Abstract]) OR (“heart*”[Title/Abstract] AND “diffusion-tensor”[Title/Abstract]) OR (“cardio*”[Title/Abstract] AND “diffusion-tensor”[Title/Abstract]) OR (“cardiac”[Title/Abstract] AND “diffusion weight*”[Title/Abstract]) OR (“heart*”[Title/Abstract] AND “diffusion weight*”[Title/Abstract]) OR (“cardio*”[Title/Abstract] AND “diffusion weight*”[Title/Abstract]) OR (“cardiac”[Title/Abstract] AND “diffusion weight*”[Title/Abstract]) OR (“heart*”[Title/Abstract] AND “diffusion weight*”[Title/Abstract]) OR (“cardio*”[Title/Abstract] AND “diffusion weight*”[Title/Abstract]) OR (“cardiac"[Title/Abstract] AND “diffusion weight*”[Title/Abstract]) OR (“heart*”[Title/Abstract] AND “diffusion weight*”[Title/Abstract]) OR (“cardio*”[Title/Abstract] AND “diffusion weight*”[Title/Abstract]) OR (“cardiac”[Title/Abstract] AND “intravoxel incoherent motion”[Title/Abstract]) OR (“heart*”[Title/Abstract] AND “intravoxel incoherent motion”[Title/Abstract]) OR (“myocard*”[Title/Abstract] AND “diffusion-tensor”[Title/Abstract]) OR (“myocard*”[Title/Abstract] AND “diffusion-tensor”[Title/Abstract]) OR (“myocard*”[Title/Abstract] AND “diffusion weight*”[Title/Abstract]) OR (“myocard*”[Title/Abstract] AND “diffusion weight*”[Title/Abstract]) OR (“myocard*”[Title/Abstract] AND “intravoxel incoherent motion”[Title/Abstract])) AND ((“adaptive clinical trial”[Publication Type] OR “case reports”[Publication Type] OR “classical article”[Publication Type] OR “clinical study”[Publication Type] OR “clinical trial”[Publication Type] OR “clinical trial protocol”[Publication Type] OR “clinical trial, phase i”[Publication Type] OR “clinical trial, phase ii”[Publication Type] OR “clinical trial, phase iii”[Publication Type] OR “clinical trial, phase iv”[Publication Type] OR “newspaper article”[Publication Type] OR “clinical trial, veterinary”[Publication Type] OR “comparative study”[Publication Type] OR “controlled clinical trial”[Publication Type] OR “evaluation study”[Publication Type] OR “guideline”[Publication Type] OR “historical article”[Publication Type] OR “interactive tutorial”[Publication Type] OR “introductory journal article”[Publication Type] OR “meta analysis”[Publication Type] OR “multicenter study”[Publication Type] OR “network meta analysis”[Publication Type] OR “observational study”[Publication Type] OR “observational study, veterinary”[Publication Type] OR “overall”[Publication Type] OR “practice guideline”[Publication Type] OR “pragmatic clinical trial”[Publication Type] OR “preprint”[Publication Type] OR “haserratumfor”[All Fields] OR “randomized controlled trial”[Publication Type] OR “randomized controlled trial, veterinary”[Publication Type] OR “review”[Publication Type] OR “scientific integrity review”[Publication Type] OR “scoping review”[Publication Type] OR “systematic review”[Filter] OR “technical report”[Publication Type] OR “validation study”[Publication Type]) AND 2005/01/01:2025/12/31[Date—Publication])) AND ((adaptiveclinicaltrial[Filter] OR casereports[Filter] OR classicalarticle[Filter] OR clinicalstudy[Filter] OR clinicaltrial[Filter] OR clinicaltrialprotocol[Filter] OR clinicaltrialphasei[Filter] OR clinicaltrialphaseii[Filter] OR clinicaltrialphaseiii[Filter] OR clinicaltrialphaseiv[Filter] OR veterinaryclinicaltrial[Filter] OR comparativestudy[Filter] OR controlledclinicaltrial[Filter] OR evaluationstudy[Filter] OR guideline[Filter] OR historicalarticle[Filter] OR interactivetutorial[Filter] OR introductoryjournalarticle[Filter] OR meta-analysis[Filter] OR multicenterstudy[Filter] OR networkmetaanalysis[Filter] OR newspaperarticle[Filter] OR observationalstudy[Filter] OR veterinaryobservationalstudy[Filter] OR overall[Filter] OR practiceguideline[Filter] OR pragmaticclinicaltrial[Filter] OR preprint[Filter] OR publishederratum[Filter] OR randomizedcontrolledtrial[Filter] OR randomizedcontrolledtrialveterinary[Filter] OR review[Filter] OR scientificintegrityreview[Filter] OR scopingreview[Filter] OR systematicreview[Filter] OR technicalreport[Filter] OR validationstudy[Filter]) AND (2005:2024[pdat]))

#### 2.1.2 Article screening

The inclusion criteria of cDMRI articles were as follows: (a) Article types, original research and reviews; (b) Publication date, from January 1, 2005 to June 30, 2024; (c) Article language, English. The exclusion criteria were as follows: The articles unrelated to cDMRI primarily consisted of studies that performed diffusion MRI examinations on participants with cardiac diseases, but for organs other than the heart (e.g., the brain).

All downloaded records were independently screened by two reviewers to exclude the articles unrelated to cDMRI. Any disagreements were resolved by a third independent reviewer. Duplicate articles retrieved from both databases were identified and manually removed by the two reviewers. As all data were directly retrieved from the databases, no ethical approval was required for this study.

### 2.2 Data analysis and visualization

Bibliometric analysis and visualization were performed using Citespace 6.2.R6, VOSviewer 1.6.20, and the R package “bibliometrix”(version 4.4.1) (https://www.bibliometrix.org).

The bursts of keywords and references were using Citespace with the parameters configured as follows: (a) Time slice from 2005 to 2024 with each slice representing one year; (b) Selection criteria defined as Top *N* = 25; (c) Pruning methods performed using the pathfinder and pruning sliced networks (22). Additionally, VOSviewer was employed to construct and analyze knowledge maps, facilitating the identification of collaborative networks. The R package “bibliometrix” was used for visualizing topic trends in cDMRI.

## 3 Results

### 3.1 Annual publications

A total of 359 articles were retrieved from WoSCC and 88 from PubMed. After screening, 367 articles met the search criteria in both databases, and only 8 articles could be searched only in PubMed rather than WoSCC. Figure 2 displayed the annual distribution of cDMRI-related publications between 2005 and 2024. Although the absolute number of publications remains relatively modest, there is a clear upward trend, reflecting increasing research interest and growing recognition of cDMRI's potential in cardiovascular imaging.

**Figure 2:**
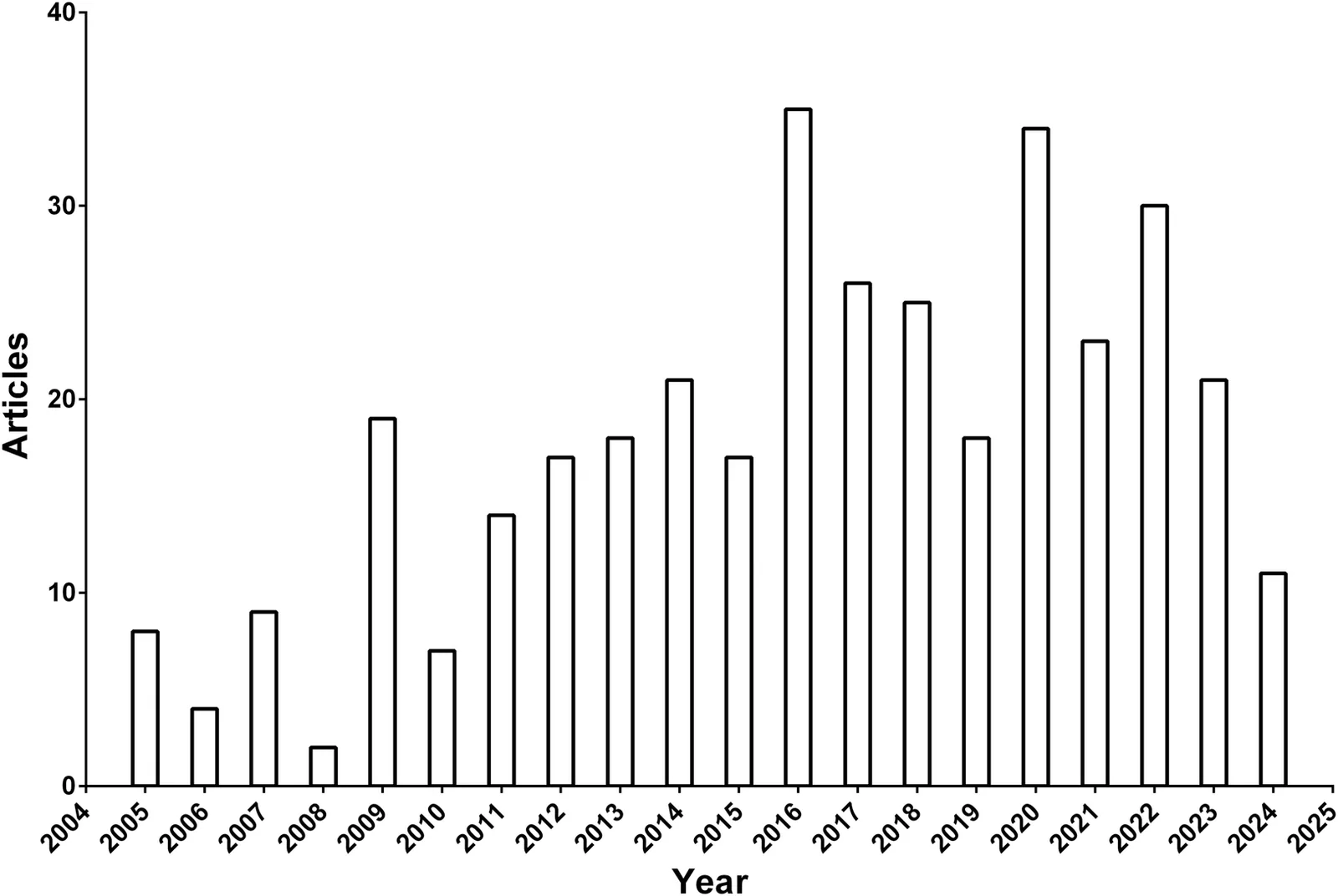
Global trend of publications on cardiac diffusion magnetic resonance imaging research in the past 20 years. The absolute number of publications remained relatively modest, indicating an upward trend.

### 3.2 Analysis of journals

Table 1 listed the top 10 most productive journals in cDMRI research. *Magnetic Resonance in Medicine* ranked first with 48 publications (IF 2023, 3.0; JCR 2023, Q2), followed by the *Journal of Cardiovascular Magnetic Resonance* (27 articles; IF 2023, 4.2; JCR 2023, Q1) and the *Journal of Magnetic Resonance Imaging* (19 articles; IF 2023, 3.3; JCR 2023, Q1).

**Table 1:** The top 10 productive journals in cardiac diffusion magnetic resonance imaging research.

Sources	Articles	Country	IF 2023	JCR 2023
Magnetic resonance in medicine	48	United States	3.0	Q2
Journal of cardiovascular magnetic resonance	27	United States	4.2	Q1
Journal of magnetic resonance imaging	19	United States	3.3	Q1
Ieee transactions on medical imaging	14	United States	8.9	Q1
Nmr in biomedicine	14	England	2.7	Q2
American journal of physiology-heart and circulatory physiology	10	United States	4.1	Q1
Medical image analysis	10	Netherlands	10.7	Q1
Ieee transactions on biomedical engineering	9	United States	4.4	Q2
Physics in medicine and biology	9	England	3.3	Q1
Plos one	9	United States	2.9	Q1

IF, impact factor; JCR, Journal Citation Reports.

Table 2 listed the top 10 most cited journals. *Magnetic Resonance in Medicine* had the highest citation count (2358 citations), followed by the *American Journal of Physiology-Heart and Circulatory Physiology* (977 citations; IF 2023, 4.1; JCR 2023, Q1) and the *Journal of Cardiovascular Magnetic Resonance* (865 citations, IF 2023, 4.2; JCR 2023, Q1). Notably, several high-impact journals featured prominently in the top 10 most cited list, including *Circulation* (804 citations, IF 2023, 35.5; JCR 2023, Q1), *Circulation Research* (646 citations; IF 2023, 16.5; JCR 2023, Q1), *Journal of the American College of Cardiology* (424 citations; IF 2023, 21.7; JCR 2023, Q1), and *Radiology* (292 citations; IF 2023, 12.1; JCR 2023, Q1).

**Table 2:** The top 10 journals with most citations in cardiac diffusion magnetic resonance imaging research.

Sources	Citations	Country	IF 2023	JCR 2023
Magnetic resonance in medicine	2358	United States	3.0	Q2
American journal of physiology-heart and circulatory physiology	977	United States	4.1	Q1
Journal of cardiovascular magnetic resonance	865	United States	4.2	Q1
Circulation	804	United States	35.5	Q1
Circulation research	646	United States	16.3	Q1
Journal of magnetic resonance imaging	461	United States	3.3	Q1
Journal of the american college of cardiology	424	United States	21.7	Q1
Ieee transactions on medical imaging	386	United States	8.9	Q1
Nmr in biomedicine	314	England	2.7	Q2
Radiology	292	United States	12.1	Q1

IF, impact factor; JCR, Journal Citation Reports.

### 3.3 Analysis of countries, institutes, and authors

Supplementary Figure S1A illustrated the international collaboration network in cDMRI research, while Supplementary Figure S1B presented the institutional collaboration network. Supplementary Table S1 ranked the top 10 most active countries, and Supplementary Table S2 identified the top authors in cDMRI research. Supplementary Figure S2A visualized the co-authorship network, while Supplementary Figure S2B mapped the relationships among between the most frequently cited authors.

### 3.4 Analysis of co-cited references

Table 3 listed the top 25 co-cited references in cDMRI, while Figure 3 illustrated the top 25 strongest citation bursts.

**Table 3:** Top 25 documents with most citations in cardiac diffusion magnetic resonance imaging research.

Paper	TC	TC per year	Normalized TC
Bayer JD, 2012, Ann Biomed Eng	346	26.62	4.85
Wu MT, 2006, Circulation	214	11.26	2.16
Helm P, 2005, Ann Ny Acad Sci	193	9.65	1.55
Helm PA, 2005, Magn Reson Med	175	8.75	1.40
Lombaert H, 2012, Ieee T Med Imaging	167	12.85	2.34
Walker JC, 2005, Am J Physiol-Heart C	166	8.30	1.33
Nielles-Vallespin S, 2017, J Am Coll Cardiol	163	20.38	5.09
Chen JJ, 2005, Am J Physiol-Heart C	152	7.60	1.22
Helm PA, 2006, Circ Res	147	7.74	1.48
Krishnamurthy A, 2013, J Comput Phys	146	12.17	3.48
Wang VY, 2009, Med Image Anal	130	8.13	2.71
Pashakhanloo F, 2016, Circ-Arrhythmia Elec	128	14.22	5.45
Ferreira PF, 2014, J Cardiovasc Magn R	127	11.55	3.36
Nielles-Vallespin S, 2013, Magn Reson Med	126	10.50	3.00
Plank G, 2009, Philos T R Soc A	125	7.81	2.61
Gurev V, 2011, Biomech Model Mechan	117	8.36	2.76
Rohmer D, 2007, Invest Radiol	117	6.50	1.82
Cao Y, 2005, Ieee T Med Imaging	111	5.55	0.89
Gamper U, 2007, Magn Reson Med	107	5.94	1.66
Wu EX, 2007, Magn Reson Med	103	5.72	1.60
Wu MT, 2009, Circ-Cardiovasc Imag	95	5.94	1.98
Yeh FC, 2017, Magn Reson Med	95	11.88	2.97
Peyrat JM, 2007, Ieee T Med Imaging	93	5.17	1.44
Jeong EK, 2005, Magnet Reson Med	91	4.55	0.73
Bayer JD, 2012, Ann Biomed Eng	346	26.62	4.85

TC, total citation.

**Figure 3:**
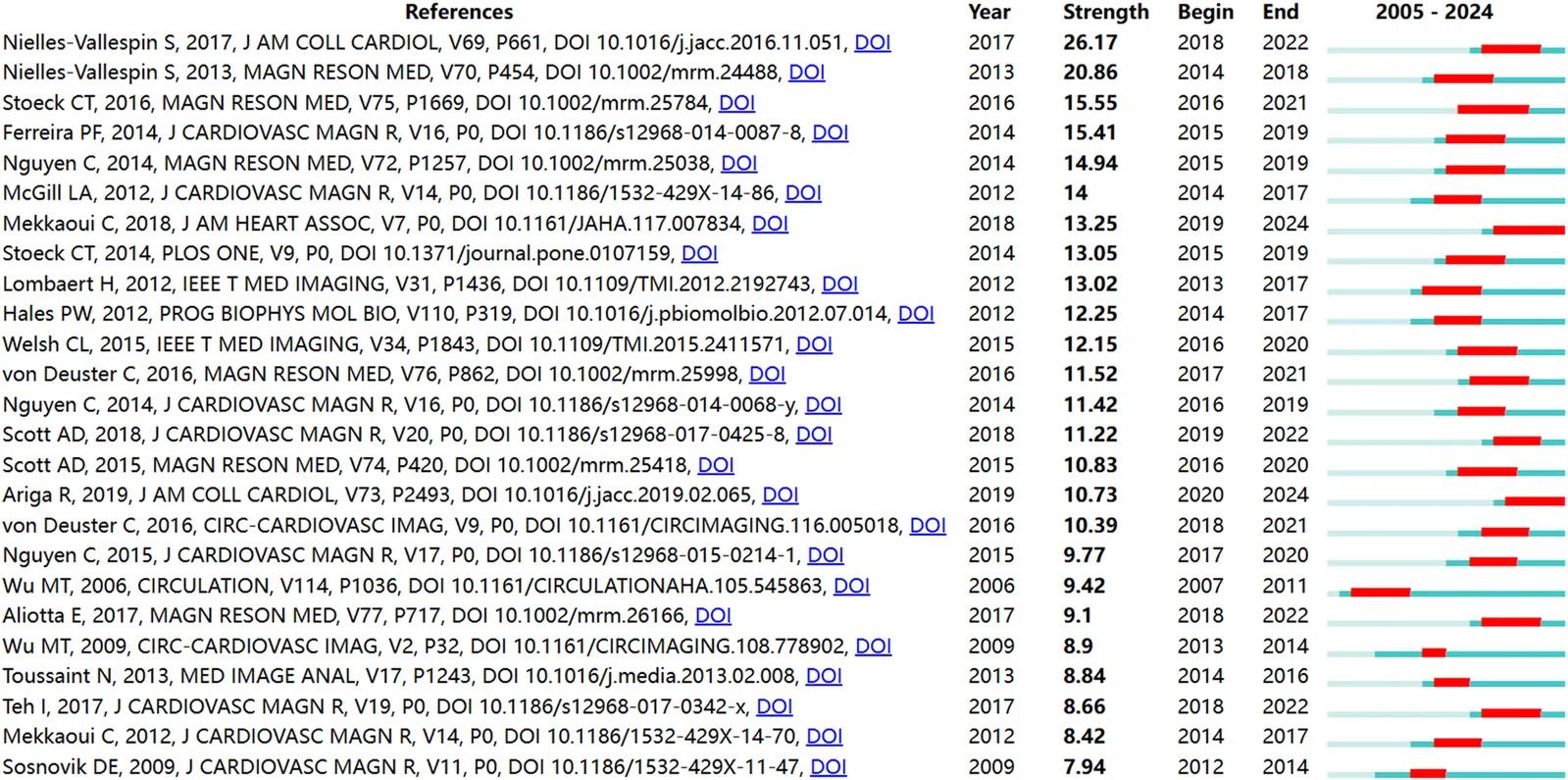
Top 25 references with the strongest citation bursts in cardiac diffusion magnetic resonance imaging research. The citation bursts of references were assessed using CiteSpace with the parameters configured as follows: Time slice from 2005 to 2024 with each slice representing one year; Selection criteria defined as Top *N* = 25; Pruning methods performed using the pathfinder and pruning sliced networks. A citation burst indicates the likelihood that the scientific community has paid or is paying special attention towards the underlying contribution. The light blue lines indicated time period from 2005 to 2024. The beginning of dark blue lines depicted when articles were published. The beginning of red segments marked the beginning of a period of burst, whereas the end of the red segment marked the end of the burst period. Citation burst strength quantified the intensity and speed of influence within cardiac diffusion magnetic resonance imaging research. It measured how rapidly the articles were cited, indicating a surge of recent academic interest or relevance.

Among the most influential studies, The 2012 article by J.D. Bayer et al. in Annals of Biomedical Engineeringranked first in total citations (346.0), citations per year (26.62), and normalized citations per year (4.85). This study proposed a novel Laplace–Dirichlet Rule Based (LDRB) algorithm for generating cDTI-based myocardial fiber orientations in computational heart models, achieving high speed, precision, and usability (23). J.D. Bayer et al. indicated that the LDRB algorithm could be applied in cardiac electrophysiology and simulations of electrical activation in heart model. In addition, Ming-Ting Wu et al. in 2006 published in *Circulation* is significant research in cDMRI, accumulating 214 total citations with an annual citation rate of 11.26 (24). This study used *in vivo* cDTI to investigate myocardial fiber remodeling after infarction, using ADC, fractional anisotropy (FA), and fiber helix angles (HA) as markers of microstructural integrity and viability (24).

The strongest citation bursts (26.17) was observed for the 2017 article by Sonia Nielles-Vallespin et al. in the Journal of the *American College of Cardiology*, with 163 total citations and an average of 20.38 citations per year (10). This study validated the ability of *in vivo* cDTI to characterize myocardial microstructure dynamics and emphasized its clinical relevance for heart disease assessment. Additionally, another high-impact study by Sonia Nielles-Vallespin et al. implemented an optimized stimulated echo acquisition mode (STEAM) imaging sequence, which was combined with a modified prospective navigator technique and a biofeedback mechanism (25), achieving the second-strongest citation bursts (20.86). The third-strongest citation burst (15.55) was recorded for Christian T. Stoeck et al., who pioneered the implementation of second-order motion-compensated spin echo (M2C) cDTI for *in-vivo* human heart imaging on a clinical MRI scanner (26).

### 3.5 Analysis of keywords and trend topics

In terms of co-occurrence keyword clustering, it could be roughly divided into 4 clusters of various colors in the present study. Cluster 1 pertained to the sequence design of cDMRI, with representative keywords including “motion compensation”, “steam”, “spin echo”, and “free-breathing”. Cluster 2 focused on the cDTI image processing aspects of cDMRI, exemplified by keywords such as “mean diffusivity”, “fractional anisotropy”, “helix angle”, “image registration”, and “image reconstruction”. Cluster 3 was centered on clinical applications in various cardiac diseases, covering keywords like “heart failure”, “dilated cardiomyopathy”, “congenital heart disease”, “ventricular arrhythmias”, and “ischemia”. Cluster 4 related to the linkage between cDMRI and other cardiac MRI techniques, represented by keywords such as “late gadolinium enhancement”, “T1 mapping”, “T2 mapping”, and “strain”.

The keyword co-occurrence map (Figure 4) and Table 4 highlighted the most frequently occurring terms in cDMRI research. “diffusion tensor imaging” ranked as the most prominent keyword, followed by “diffusion-weighted imaging” and “cardiac fiber”. The occurrences (191), total link strength (370), and centrality (1.00) of “diffusion tensor imaging” underscored its central role in the field. The “diffusion weighted imaging”, “cardiac fiber” and “myocardial infarction” also demonstrated substantial influence, each with centrality values exceeding 0.10. Notably, key research areas such as “myocardial infarction”, “cardiac microstructure”, “hypertrophic cardiomyopathy”, “deep learning”, “motion compensation”, “compressed sensing” and “cardiac electrophysiology” were among the top 20 co-occurrence keywords, all with centralities above 0.05.

**Figure 4:**
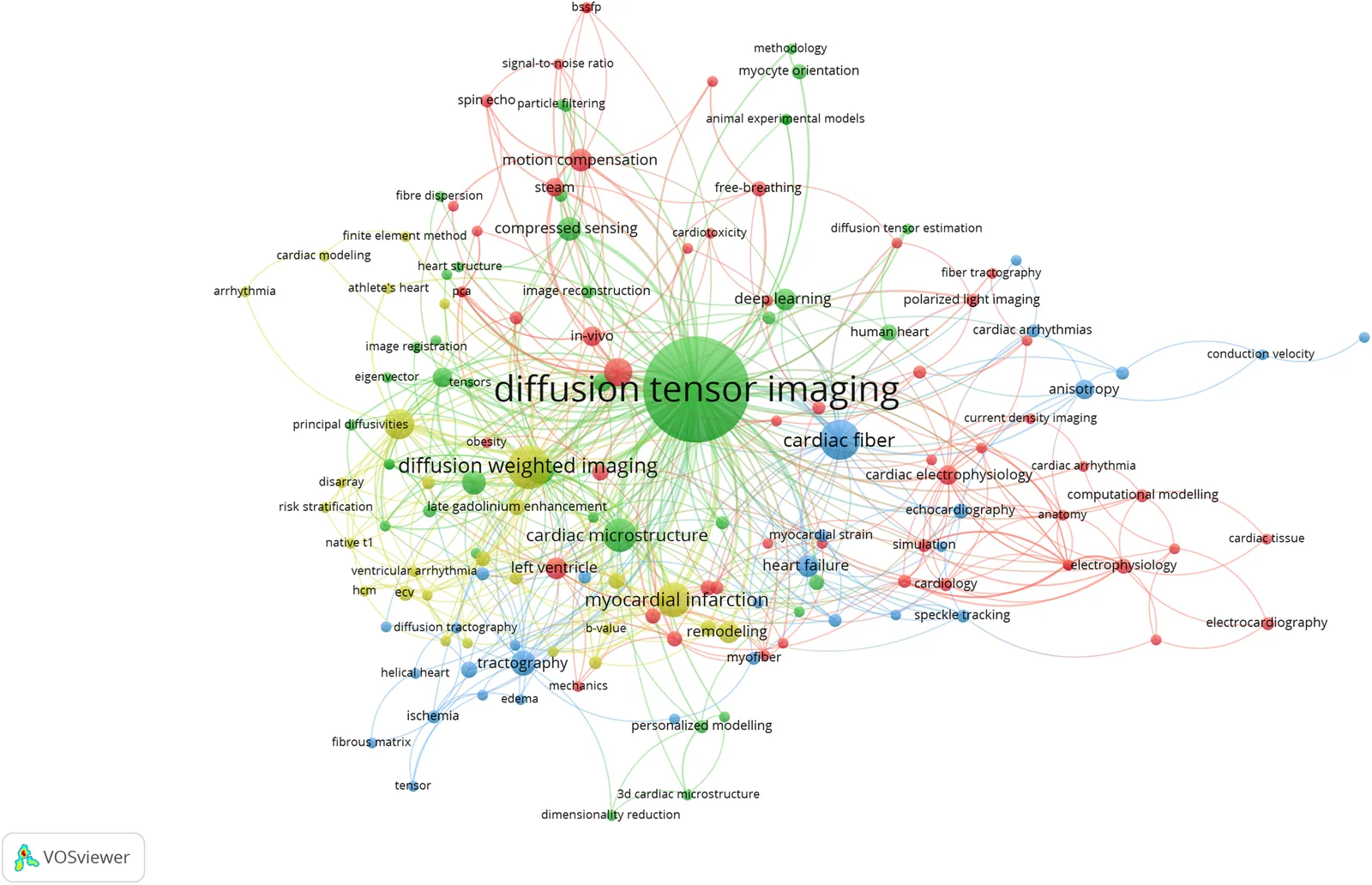
The network of keywords. The VOSviewer was used to conduct the keyword co-occurrence analysis. The width of links between nodes represented the strength of association between author keywords, with thicker links indicating stronger relationships. The size of each node corresponded to the occurrence frequency of keywords, where larger node size denoted keywords that appeared more frequently. Nodes sharing the same color belonged to the same cluster.

**Table 4:** Top 20 keywords in cardiac diffusion magnetic resonance imaging research.

Keyword	Occurences	Total link strength	Centrality
Diffusion tensor imaging	191	280	0.98
Diffusion weighted imaging	32	63	0.16
Cardiac fiber	28	41	0.21
Myocardial infarction	21	48	0.1
Cardiac microstructure	20	49	0.05
Hypertrophic cardiomyopathy	16	35	0.09
Intravoxel incoherent motion	14	22	0.04
Helix angle	13	34	0.12
Tractography	11	33	0.01
Fractional anisotropy	10	27	0.06
motion compensation	9	21	0.06
Compressed sensing	8	13	0.05
Deep learning	8	14	0.04
Heart failure	8	14	0.05
Remodeling	8	20	0
Anisotropy	7	9	0.01
Cardiac electrophysiology	7	9	0.07
*In-vivo*	7	20	0.03
Mean diffusivity	7	23	0.02
Laminar structure	6	17	0.01

The burst keyword analysis (Figure 5) revealed the top 15 keywords with the strongest citation bursts. The cardiac “diffusion tensor imaging” exhibited the strongest citation bursts (6.8) from 2022 to 2024, emphasizing its recent surge in research attention. The “cardiac microstructure” and “spin echo” followed as the second and third strongest citation bursts, respectively. In addition, the “deep learning” demonstrated sustained growth, with a burst strength of 3.93 beginning in 2020 and continuing through 2024, indicating its increasing impact in cDMRI research.

**Figure 5:**
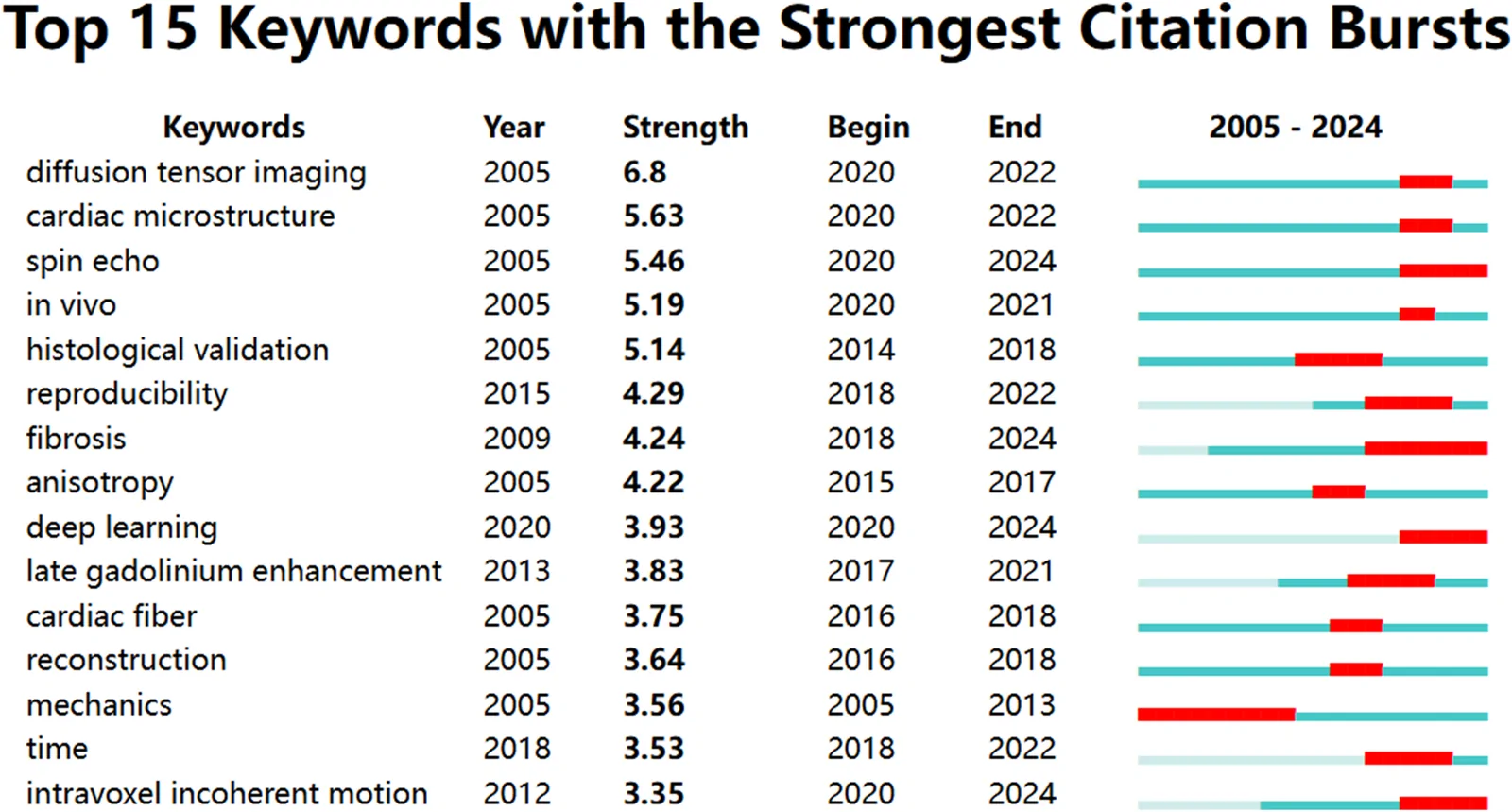
Top 15 keywords with the strongest citation bursts in cardiac diffusion magnetic resonance imaging research. The citation bursts of keywords were assessed using CiteSpace with the parameters configured as follows: Time slice from 2005 to 2024 with each slice representing one year; Selection criteria defined as Top *N* = 25; Pruning methods performed using the pathfinder and pruning sliced networks. A citation burst indicates the likelihood that the scientific community has paid or is paying special attention towards the underlying contribution. The light blue lines indicated time period from 2005 to 2024. The beginning of dark blue lines depicted when keywords have attracted considerable attention. The beginning of red segments marked the beginning of a period of burst, whereas the end of the red segment marked the end of the burst period. Citation burst strength of keywords acted as indicators of rapidly evolving topics and interests within a particular discipline, highlighting where significant advancements and scholarly attention were concentrated. Identifying burst keywords allowed researchers to stay informed about cutting-edge developments.

The trend topics analysis (Figure 6) further identified “deep learning” as an emerging focus since 2022, marking it as a major research frontier in cDMRI. In addition, “motion compensation”, “compressed sensing” and “cardiac electrophysiology” have been recognized as significant evolving topics, reflecting the continued advancement of cDMRI techniques.

**Figure 6:**
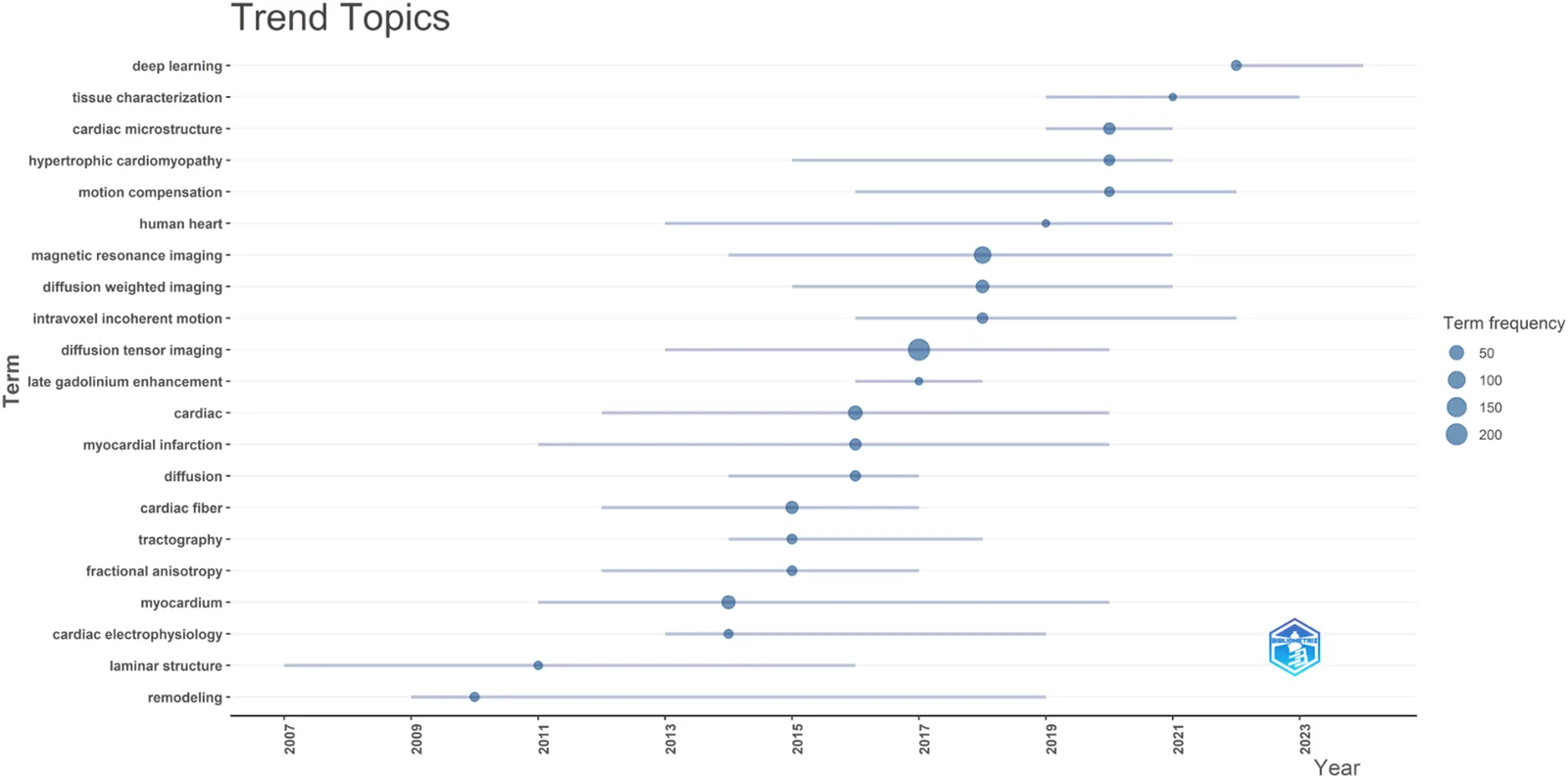
The trend topics in cardiac diffusion magnetic resonance imaging research. The R package “bibliometrix” was used to visualize topic trend maps in this study. The results indicate that “deep learning” has been a trending topic based on author keywords in recent years. The plot illustrated the evolving focus within the field of cardiac diffusion magnetic resonance imaging from 2005 to 2024. The bubble size of each keyword indicated its frequency in cardiac diffusion magnetic resonance imaging. The light blue lines for these keywords could provide insight into the consistency and distribution of their usages across the analyzed articles.

## 4 Discussion

In the study, we conducted a bibliometric analysis of cDMRI research from 2005 to 2024 using CiteSpace, the R package “bibliometrix,” and VOSviewer. We summarized the general information, research hotspots, and future trends in cDMRI research. Figure 7 provided a graphical summary of the main bibliometric findings and research trends.

**Figure 7:**
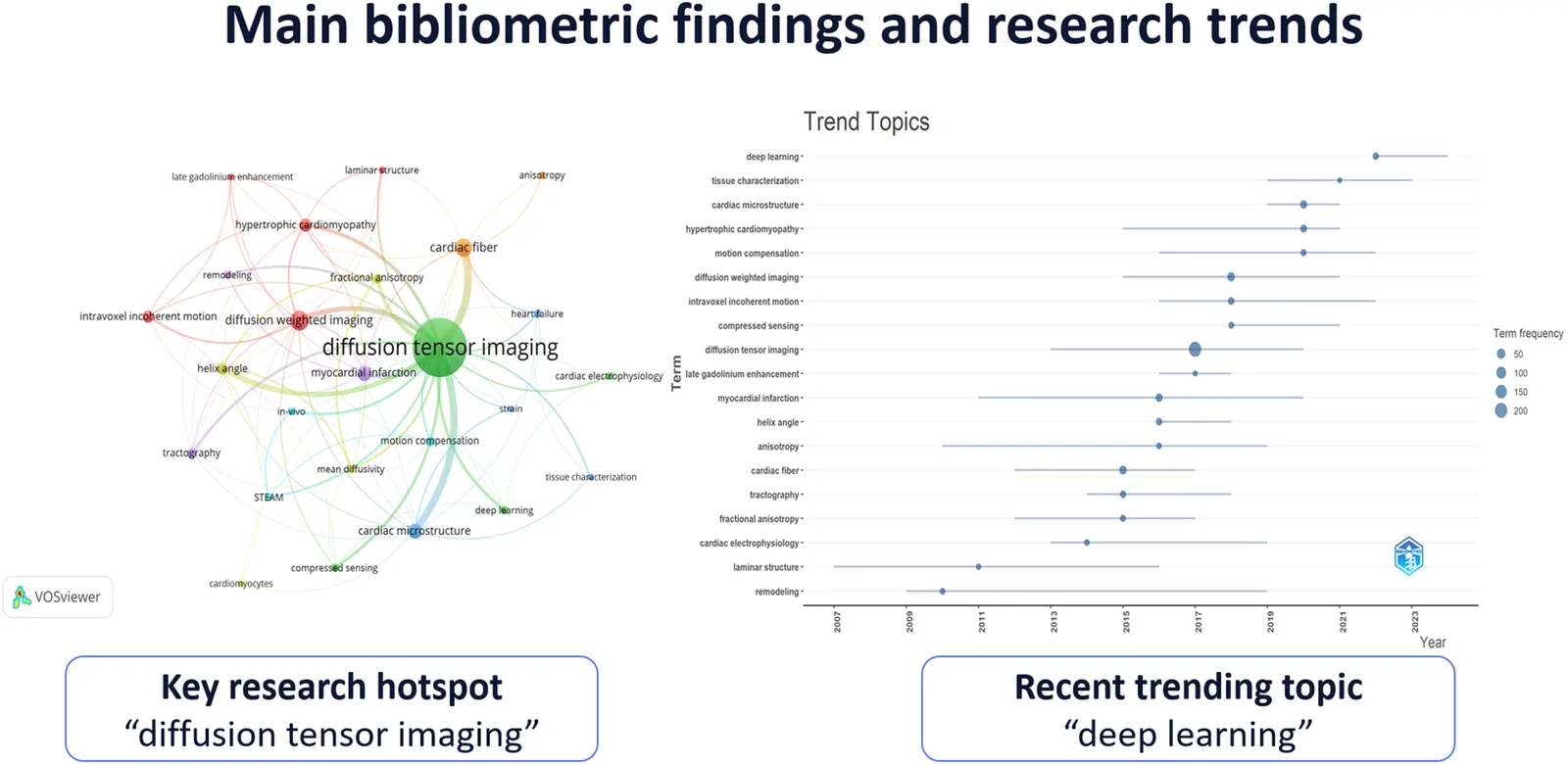
The graphical summary illustrating the main bibliometric findings and research trends. The “Diffusion tensor imaging” emerged as the most prominent keyword, with the highest occurrence count, indicating that it represents a key research hotspot. Trend topic analysis further identified “deep learning” as an emerging focus in cardiac diffusion magnetic resonance imaging research since 2022.

### 4.1 General information

Based on the systematic and comprehensive bibliometric analysis of cDMRI research from 2005 to 2024, cDMRI has been confirmed as a valuable noninvasive tool and a novel direction for assessing cardiac microstructure. The annual distribution of publications indicated a clear upward trend of cDMRI articles, reflecting increasing research interest and growing recognition of cDMRI's potential in cardiovascular imaging. Notably, cDMRI-related articles have been published in a variety of high-impact journals, further underscoring the significant research value of this field. Circulation (IF 2023, 35.5; JCR 2023, Q1), Journal of the American College of Cardiology (IF 2023, 21.7; JCR 2023, Q1) and Circulation Research (IF 2023, 16.5; JCR 2023, Q1) are the journals that stand as leading global resources for impactful cardiovascular research, delivering essential peer-reviewed articles and crucial clinical practice guidelines. Radiology (IF 2023, 12.1; JCR 2023, Q1), Journal of Cardiovascular Magnetic Resonance (IF 2023, 4.2; JCR 2023, Q1) and Journal of Magnetic Resonance Imaging (IF 2023, 3.3; JCR 2023, Q1) publish high-quality articles on the design, development, manufacture, and evaluation of CMR methods applied to the cardiovascular system. Publication in these high-impact journals reinforced the academic standing and clinical relevance of cDMRI.

The clinical application and technical developments of *in-vivo* cDTI were recognized as current research hotspots, as evidenced by the high-impacted articles and cluster analysis of keywords. The articles by Sonia Nielles-Vallespin et al. in 2017 exhibited the strongest citation burst, which validated the ability of *in-vivo* cDTI to characterize myocardial microstructure dynamics and underscored its clinical relevance for heart disease assessment. Ming-Ting Wu et al. in 2006, the most cited work in cDMRI, used *in-vivo* cDTI indices as markers of microstructural integrity and viability investigate myocardial fiber remodeling after infarction. In the article with second-strongest citation burst, Sonia Nielles-Vallespin et al. implemented a novel STEAM method, which was combined with a modified prospective navigator technique and a biofeedback mechanism (25). Christian T. Stoeck et al. pioneered the implementation of M2C cDTI for *in-vivo* human heart imaging on a clinical MRI scanner, and their study exhibited the third-strongest citation burst. Among the most influential studies, J.D. Bayer et al. in 2012 in *Annals of Biomedical Engineering* ranked first in total citations, total citation per year and normalized total citations per year. This study introduced a novel LDRB algorithm, which was well suited for cardiac electrophysiology studies and simulations of electrical activation in heart models (23). In terms of co-occurrence keyword clustering, approximately four clusters could be distinguished, corresponding to sequence design, cDTI image processing, clinical applications in various cardiac diseases, and the linkage between cDMRI and other CMR techniques.

Notably, “deep learning” has emerged as a growing research focus since 2022, because integrating deep learning may improve cDMRI scanning efficiency. In addition, the “deep learning” demonstrated sustained growth, with a burst strength of 3.93 spanning from 2020 to 2024, underscoring its increasing impact in cDMRI research. Thereby, deep learning enhances the clinical utility of cDMRI and strengthens the potential of preclinical techniques based on cDMRI. Moreover, “cardiac electrophysiology” was also identified as a significant evolving topic, reflecting the continued progress in cDMRI-based preclinical approaches. J.D. Bayer et al. in 2012 in *Annals of Biomedical Engineering* led in total citations, annual citations, and normalized annual citations. Their proposed LDRB algorithm is ideally applicable to cardiac electrophysiology and electrical activation simulations in heart models.

### 4.2 Research hotspots

A bibliometric analysis commonly utilizes frequent keywords, citation and co-citation analyses of references to determine notable literature and evaluate research progression (22). Articles with a high number of citations typically represent high-quality research characterized by substantial innovation and remarkable influence within their respective fields.

As revealed by the frequent keywords and highly cited studies, the “diffusion tensor imaging” ranked as the most prominent keyword with the most occurrences (191.0) and strongest citation bursts (6.8). Analysis of highly cited studies in cardiac “diffusion tensor imaging revealed” that: the research hotspots in cDTI appeared to consist of three main areas, including clinical application of cDTI, sequence design of cDTI, and cardiac electrophysiology based on cDTI.

#### 4.2.1 Clinical application of cDTI

The results have demonstrated that, exploring cDTI's clinical applications is probably the main direction of cDMRI. The articles by Sonia Nielles-Vallespin et al. in 2017 had the strongest citation burst, which validated the ability of *in-vivo* cDTI to characterize myocardial microstructure dynamics and emphasized its clinical relevance for heart disease assessment (10). Ming-Ting Wu et al. in 2006, the study with most citations in cDMRI, used *in-vivo* cDTI as markers of microstructural integrity and viability to investigate myocardial fiber remodeling after infarction. In addition, the presence of “myocardial infarction” (MI) and “hypertrophic cardiomyopathy” (HCM) among the top 10 keywords highlights that cDMRI has become a valuable diagnostic and prognostic tool, particularly in MI and HCM.

The *in-vivo* evaluation of myocardial microstructural dynamics has been a useful tool for assessing *in-vivo* myocardial microstructure (1–3), which can be evaluated by cDTI (9–12). The sheetlets reorientation mechanism, quantified by sheetlet angles (E2A), plays a crucial role in left ventricular wall thickening (10). Nielles-Vallespin et al. validated cDTI-derived microstructure indices *in vivo* and observed significant variations between systole and diastole, with E2A increasing from 18° (diastole) to 65°(systole) (10). In addition, McGill et al. indicated that E2A values varied across different myocardial slices in both phases, further supporting its role in cardiac function assessment (27). Apart from sheetlet dynamics, other cDTI parameters such as helical angle (HA), fractional anisotropy (FA), and mean diffusivity (MD) provide insights into cardiac fiber orientation (9, 27). McGill et al. indicated that FA decreased from 0.56(diastole) to 0.47 (systole), while MD decreased from 1.11 × 10^3^ mm^2^/s to 0.93 × 10^3^ mm^2^/s (27), reflecting the structural reorganization during cardiac contraction and relaxation.

The bibliometric analysis identified MI as one of the major clinical application areas of cDTI, supported by several highly cited studies demonstrating its diagnostic and prognostic value. The cDTI effectively detects MI in both infarcted and non-infarcted myocardium, aligning with histological findings (11, 28, 29). In Wu et al. reported increased MD, decreased FA, and decreased HA in infarct-adjacent zones, with sequential changes over time (29). Das et al. found that FA in acute infarcted myocardium was an independent predictor of 3-month left ventricular ejection fraction, demonstrating its prognostic value (11). Longitudinal studies indicated that FA and E2A changes predicted adverse LV remodeling over a 12-month follow-up (28).

HCM was recognized as another major clinical application areas of cDTI revealed by the bibliometric analysis. The HCM patients exhibited abnormal E2A values, particularly in diastole, where E2A remained elevated in hypertrophic regions (9, 10). Ferreira et al. found that E2A values in HCM patients were elevated in both systole and diastole, with abnormally high angulation retained in hypertrophic segments (9). Ariga et al, reported significantly reduced FA in HCM patients, with mid-wall FA disruption linked to ventricular arrhythmias (30).

However, the lack of standardization and interpretation of cDMRI protocols remains a major obstacle to its clinical adoption. Table 5 showed that, the diversity of cDMRI sequences, acquisition parameters, and MRI hardware systems across previous studies has compromised the comparability and interpretability of cDTI indices. Factors such as sequence type (e.g., STEAM vs. M2C), acquisition parameters (e.g., acquisition resolution, b-values, or cardiac phases), and MRI hardware (e.g., manufacturer, field strength, and coil equipment) all influence the SNR of cDMRI images. It has been demonstrated that SNR significantly affects derived cDMRI parameters, including ADC, FA, MD, HA, and E2A. Additionally, the frequent use of in-house post-processing software in prior studies has introduced variability in cDTI indices, highlighting the need for a standardized analysis tool for cDTI. Furthermore, large-scale, multi-center, multi-vendor longitudinal studies are urgently required to confirm the clinical reproducibility and applicability of cDMRI. Aside from Ferreira et al. and Huang et al., no other studies had a sample size larger than 500 (31, 32). After that, the normal reference ranges for cDMRI indices can be establish, and thereby advance the clinical translation of cDMRI. Therefore, several recommendations can be offered to guide future research based on our findings: First, cDMRI protocols adapted to various MRI hardware systems should be standardized. Second, a standardized analysis tool for cDTI should be developed. Third, a large-scale, multi-center, multi-vendor longitudinal study should be performed. Fourth, the SNR of cDMRI images should be reported, and corresponding normal reference ranges for cDMRI indices should be established.

**Table 5:** Summary of technical parameters of cDMRI focused on *in-vivo* human hearts.

Reference	Sequences	TR (ms)	TE (ms)	Resolution (mm×mm×mm)	B-values (s/mm^2^)	Number. diffusion directions	Bandwidth (Hz/pixel)	Field strength (Tesla)	Respiratory compensation	FOV (mm×mm)	Cardiac phase	Vendor
Wu et al. (24)	STEAM	2 R-R	42	1.9 × 1.9 × 8	0, 300	7	–	1.5	breath-hold	240 × 240	mid-systole	Cvi, Signa, General Electric
Nielles-Vallespin et al. (10)	STEAM	2 R-R	30	2.0 × 2.0 × 8	150, 500	6	1,838	3.0	breath-hold	320 × 120	end-diastole	Magnetom Skyra, Siemens
											end-systole	
Khalique et al. (45)	STEAM	2 R-R	25	2.8 × 2.8 × 8	150, 600	6	–	3.0	breath-hold	–	end-diastole	Magnetom Skyra, Siemens
											end-systole	
Deuster et al. (12)	STEAM	2 R-R	18	2.5 × 2.5 × 8	0, 350	10	–	1.5	breath-hold	309 × 129	end-systole	Achieva, Philips
											mid-diastole	
Wu et al. (29)	STEAM	2 R-R	42	1.9 × 1.9 × 8	0, 300	7	–	1.5	breath-hold	240 × 240	end-diastole	CVi, General Electric
											end-systole	
Das et al. (28)	M2C	3 R-R	89	2.2 × 2.3 × 8	100, 200, 500	12		3.0	free-breathing	238 × 238	mid-systole	Achieva, Philips
An et al. (46)	ssEPI	2.9	1.6	2.0 × 2.0 × 8	0, 10, 50, 100, 150, 200, 250, 500, 800	–	–	3.0	free-breathing	300 × 300	–	Ingenia, Philips
Li et al. (47)	ssEPI	857	51	3.5 × 3.5 × 8	0, 20, 50, 80, 100, 120, 200, 300, 500	–	–	3.0	free-breathing	300 × 300	–	HDxt, General Electric
Wu et al. (48)	M2C	800	77	1.2 × 1.2 × 10	0, 350	3	1,449	3.0	free-breathing	230 × 230	end-diastole	Ingenia, Philips
											end-systole	
Sharifian et al. (49)	ssEPI	90	54	1.5 × 1.5 × 10	0, 100, 150	3	2,220	1.5	breath-hold	–	systole	Magnetom Avanto, Siemens
Joy et al. (50)	M2C	3 R-R	77	2.3 × 2.3 × 8	100, 450	30	–	3.0	free-breathing	320 × 121	end-systole	Prisma, Siemens
Das et al. (51)	M2C	3 R-R	77	2.3 × 2.3 × 8	100, 450	30	–	3.0	free-breathing	320 × 121	30% systole	Prisma, Siemens
Wu et al. (52)	ssEPI	3 R-R	61	3.5 × 3.5 × 8	0, 10, 20, 50, 100, 150, 200, 250, 500, 800	3	2,371	3.0	free-breathing	400 × 350	end-systole	Ingenia, Philips
Deux et al. (53)	ssEPI	3,000	78	1.7 × 1.7 × 10	0, 50	3	1,386	1.5	free-breathing	320 × 320	mid-diastole	Magnetom Avanto, Siemens
Li et al. (54).	ssEPI	–	–	–	–	–	–	3.0	–	–	–	HDxt, General Electric
Shi et al. (7)	ssEPI	4,000	60	1.8 × 1.8 × 7	0, 10, 50, 100, 250, 500	3	–	3.0	free-breathing	400 × 400	–	Ingenia, Philips
Wu et al. (55)	M2C	4.1	2.0	1.2 × 1.2 × 10	0, 350	3	1,449	3.0	free-breathing	230 × 230	end-diastole	Ingenia, Philips
											end-systole	
Delattre et al. (56)	ssEPI	110	56	2.6 × 2.6 × 6	0, 15,30, 45, 60, 75, 90, 105, 120, 250, 350,450, 550	3	–	1.5	breath-hold	236 × 420	–	Magnetom Avanto, Siemens
Rapacchi et al. (57)	ssEPI	2 R-R	51	2.6 × 2.6 × 6	0, 50, 100	3	–	1.5	free-breathing	416 × 333	diastole	Magnetom Avanto, Siemens
Ferreira et al. (9)	STEAM	2 R-R	23	2.7 × 2.7 × 8	0, 350	6	2,442	3.0	breath-hold	360 × 150	systole, diastole	Skyra, Siemens
Gotschy et al. (58)	M2C	3 R-R	76	2.5 × 2.5 × 8	100, 450	9	–	1.5	free-breathing	230 × 105	mid-systole	Achieva, Philips
Kociemba et al. (59)	ssEPI	3,000–4,000	78	2.0 × 1.6 × 10	50, 100, 200	3	1,736	1.5	free-breathing	380 × 310	–	Magnetom Avanto, Siemens
McGill et al. (27)	STEAM	2 R-R	23	2.8 × 2.8 × 8	0, 350	6	2,442	3.0	breath-hold	360 × 135	systole, diastole	Magnetom Skyra, Siemens
McGill et al. (60)	STEAM	2 R-R	23	2.7 × 2.7 × 8	0, 350	6	2,442	3.0	breath-hold	360 × 135	end-systole	Magnetom Skyra, Siemens
Nguyen et al. (8)	M2C	6 R-R	8.4	2.1 × 2.1 × 6	0, 400	3	–	3.0	free-breathing	–	–	Magnetom Verio, Siemens
Nguyen et al. (6)	M2C	4.1	2.0	1.6 × 1.6 × 8	0, 350	3	–	1.5	free-breathing	–	end-diastole	Magnetom Avanto, Siemens
											end-systole	
Scott et al. (61)	STEAM	2 R-R	25	2.8 × 2.8 × 8	0,450	6	2,442	3.0	breath-hold	–	end-diastole	Magnetom Skyra, Siemens
	M2C		76						free-breathing		end-systole	
Sharrack et al. (62)	M2C	3 R-R	89	2.2 × 2.3 × 8	100, 200, 500	12	–	3.0	free-breathing	238* 238	mid-systole	Achieva, Philips
Tunnicliffe et al. (63)	STEAM	2 R-R	22	2.8 × 2.8 × 8	15,350	6	2,442	3.0	breath-hold	360 × 135	end-diastole	Magnetom Skyra, Siemens
											end-systole	
Abulaiti et al. (64)	ssEPI	–	–	–	0, 50, 100, 200, 400, 600	3	–	3.0	-	–	end-diastole	Magnetom Skyra, Siemens
											mid-diastole	
An et al. (65)	ssEPI	2.9	1.6	2.0 × 2.0 × 8	0, 200,800	3	–	3.0	free-breathing	300 × 300	–	Ingenia, Philips
An et al. (66)	ssEPI	2.9	1.6	2.0 × 2.0 × 8	0, 10, 50, 100, 150, 200, 250, 500, 800	3	1,210	3.0	free-breathing	300 × 300	–	Ingenia, Philips
Chen et al. (67)	M2C	2,000	80	3.0 × 3.0 × 8	0, 100, 450	3	–	3.0	free-breathing	310 × 310	mid-systole	Ingenia CX, Philips
				2.5 × 2.5 × 8								
				2.0 × 2.0 × 8								
Das et al. (68)	M2C	3 R-R	89	2.3 × 2.3 × 8	100, 200, 500	18	–	3.0	free-breathing	238 × 238	mid-systole	Achieva TX, Philips
Das et al. (69)	M2C	3 R-R	89	2.2 × 2.3 × 8	100, 200, 500	18	–	3.0	free-breathing	238 × 238	mid-systole	Achieva TX, Philips
Ferreira et al. (31)	STEAM	2 R-R	23	2.8 × 2.8 × 8	150, 600	6	–	3.0	breath-hold	360 × 135	end-diastole	Magnetom Skyra, Siemens
											end-systole	Magnetom Vida, Siemens
Laissy et al. (70)	ssEPI	3,000–4,000	60–80	1.7 × 3.4 × 8	250, 300, 350, 400, 450, 500	3	–	1.5	breath-hold	440 × 440	end-diastole	Signa HDxt Twinspeed, General Electric
Mou et al. (71)	ssEPI	857	51	3.6 × 2.7 × 8	0, 20,50, 80,100, 120,200, 300, 500	3	–	3.0	free-breathing	350 × 350	end-diastole	HDxt, General Electric
Wu et al. (72)	ssEPI	800	77	1.2 × 1.2 × 10	0, 20	3	1,449	3.0	free-breathing	280 × 280	end-diastole	Ingenia, Philips
Xiang et al. (73)	ssEPI	300	56	3.5 × 3.5 × 8	0, 50, 100, 200, 400, 800	3	–	3.0	free-breathing	–	–	Magnetom Skyra, Siemens
Zhang et al. (74)	ssEPI	2 R-R	68	3.13 × 3.41 × 10	0, 10, 25, 50,75, 100, 200, 300, 400	6	–	3.0	free-breathing	260 × 200	end-diastole	Ingenia, Philips
Ariga et al. (30)	STEAM	2 R-R	22	2.8 × 2.8 × 8	15, 350	6	2,442	3.0	breath-hold	360 × 135	diastole	TIM Trio, Siemens
McGill et al. (75)	STEAM	2 R-R	22	2.8 × 2.8 × 8	150, 750	6	–	3.0	breath-hold	360 × 135	end-systole	Magnetom Skyra, Siemens
Das et al. (11)	M2C	3 R-R	89	2.20 × 2.27 × 8	100, 200, 500	12	–	3.0	free-breathing	238 × 238	mid-systole	Achieva, Philips
Mekkaoui et al. (76)	STEAM	2 R-R	34	2.5 × 2.5 × 8	0, 500	10	–	3.0	free-breathing	360 × 200	mid-systole	Magnetom Skyra, Siemens
Moulin et al. (4)	M2C	5 R-R	43	2.7 × 2.7 × 6	0, 200	3	2,442	1.5	free-breathing	220 × 350	mid-systole	Magnetom Aera, Siemens
Potet et al. (5)	ssEPI	3,000	78	1.7 × 2.2 × 10	0, 50	3	1,385	1.5	free-breathing	320* 320	mid-diastole	Magnetom Avanto, Siemens
Phipps et al. (42)	M2C	12 R-R	75	2.5 × 2.5 × 8	50, 500	12	–	3.0	free-breathing	–	–	Prisma, Siemens
Wu et al. (77)	M2C	4.1	2.0	1.2 × 1.2 × 10	0, 350	3	1,449	3.0	free-breathing	230 × 230	end-diastole	Ingenia, Philips
											end-systole	
Xiang et al. (78)	ssEPI	220	67	3.5 × 3.5 × 8	0, 20, 60, 100, 150, 200, 600	3	–	3.0	free-breathing	306 × 230	–	Magnetom Skyra, Siemens
Khalique et al. (36)	STEAM	18 R-R	25	2.8 × 2.8 × 8	150, 450	6	–	3.0	breath-hold	–	mid-systole	Magnetom Skyra, Siemens
	M2C	16 R-R	76								mid-diastole	
Nielles-Vallespin et al. (25)	STEAM	2 R-R	23	2.7 × 2.7 × 8	0, 350	6	2,442	3.0	free-breathing	–	end-systole	Magnetom Skyra, Siemens
									breath-hold			
Stoeck et al. (26)	M2C	3 R-R	73	2.7 × 2.7 × 6	0, 450	10	–	1.5	free-breathing	230 × 98	mid-systole	Achieva, Philips
Stoeck et al. (37)	STEAM	3 R-R	39	2.5 × 2.5 × 10	100, 200, 400, 600	3	–	1.5	free-breathing	230 × 100	diatole	Achieva, Philips
	M2C	6 R-R	68								systole	
von Deuster et al. (33)	STEAM	1,000	31	2.8 × 2.8 × 8	100, 450	12	–	1.5	breath-hold	230 × 98	mid-systole	Achieva, Philips
	M2C	1,000	70									

cDMRI, cardiac Diffusion Magnetic Resonance Imaging; TR, repetition time; TE, echo time; FOV, field of view; STEAM, stimulated echo acquisition mode; M2C, second-order motion-compensated spin echo; ssEPI, single-shot echo planar imaging; R-R, R-R interval.

#### 4.2.2 Sequence design of cDTI

As revealed by the references with strong citation bursts, innovations in sequence design have played a pivotal role in the development of cDTI. Sonia Nielles-Vallespin et al. implemented a novel STEAM method that incorporated a modified prospective navigator technique and a biofeedback mechanism (25). Their article achieved the second-strongest citation burst (20.86). The third-strongest citation bursts (15.55) was recorded for Christian T. Stoeck et al, who pioneered the implementation of M2C cDTI for *in-vivo* human heart imaging on a clinical MRI scanner (26). In addition, the keywords “motion compensation” and “compressed sensing” presented in the top 20 keywords. Also, it has turned out that “Magnetic Resonance in Medicine” the most productive and highly cited journal, within which the sequence optimization stands out as an important research focus.

The cDMRI has been challenging to probe in *in-vivo* hearts because of bulk motion and myocardial strain during diffusion encoding. As revealed by the references with strong citation bursts, sequence design has been the main direction to overcome these drawbacks in *in-vivo* cDMRI. In the article with second-highest burst strength, Sonia Nielles-Vallespin et al. implemented a novel STEAM method, which was combined with a modified prospective navigator technique and a biofeedback mechanism (25). Their comparison of initial and repeat scans demonstrated good reproducibility for both multiple breath-hold and navigator techniques. The proposed method showed potential for free-breathing acquisition using STEAM and delivering high-resolution in-vivo 3D cDTI tractography of the whole heart. However, the main disadvantages of STEAM are lower Signal-to-Noise Ratio (SNR) and acquisition efficiency (3). In the article with third-highest burst strength, Stoeck et al. proposed the M2C sequence to improve the SNR and acquisition efficiency (33). It commonly uses second-order motion compensation to address signal loss from cardiac motion. It offers higher SNR and acquisition efficiency compared to STEAM (26, 33), the growing interest has been found in recent studies (4, 11). Unlike STEAM, M2C acquires diffusion data in one heartbeat, offering higher acquisition efficiency, but it requires high-performance gradient systems (34, 35). M2C is better suited for free-breathing acquisition, arrhythmias patients, and whole heart coverage (3). However, probably due to differences in motion sensitivity and longer echo time, M2C performs better in the systole phase (36, 37), while STEAM is more reliable in the diastole phase (36, 37). Hence, the M2C and STEAM each possess distinct strengths and limitations.

#### 4.2.3 Cardiac electrophysiology based on cDTI

Notably, the cardiac electrophysiology based on cDTI is probably one of the most important direction in the future based on the current evidences. J.D. Bayer et al. in 2012 in *Annals of Biomedical Engineering* led in total citations, annual citations, and normalized annual citations with a value of 346.0, 26.62, 4.85, respectively. Their proposed LDRB algorithm is ideally applicable to cardiac electrophysiology and electrical activation simulations in heart models (23). In addition, the keywords “diffusion tensor imaging”, “cardiac electrophysiology”, “cardiac fiber” and “cardiac microstructure” appeared in the top 20 keywords in cDMRI research. cDTI provides critical insights into myocardial fiber orientation, which is essential for electrical activation and contraction of the heart (17, 38).

Arrhythmic risk assessment is crucial for cardiological evaluation and requires advanced diagnostic methods to identify which patients are at high risk of structural heart disease and sudden cardiac death (39). Cardiac electrophysiology studies employing simulations of electrical activation in cDTI-based heart models have been recognized as an important direction for arrhythmic risk assessment. J.D. Bayer et al. proposed a novel LDRB algorithm for producing cDTI-based myocardial fiber orientation in computational heart models with speed, precision, and high usability (23). The high quality of cDTI-based myocardial fiber orientation enables the improvement of the precision of electrical activation simulations in these models. Moreover, the finite element models of cardiac electrophysiology have been constructed using ex-vivo cDTI data (13). These models analyze electrical activity by segmenting the ventricles, generating finite element meshes, and simulating electrical properties (13). Recent studies have also explored new computational frameworks for cardiac electrophysiology simulations, aiming to enhance our understanding of conduction patterns and arrhythmogenesis (17, 40).

However, the cardiac electrophysiology simulations based on cDTI have yet to be implemented in *in-vivo* human studies, due to lower SNR, extended acquisition time, and limited spatial resolution. Future advancements in high-performance gradient systems and motion-robust acquisition techniques may enable electrophysiological modeling in *in-vivo* human hearts. Based on our findings, several recommendations can be offered to guide future cDMRI research: First, cDTI data used for cardiac electrophysiology models should be acquired from high-performance MRI hardware systems. Second, future applications of cardiac electrophysiology models will benefit from optimized cDMRI sequences with higher SNR and shorter acquisition times.

### 4.3 Future trends

Bibliometric trend topic analysis offers a valuable means of anticipating future research directions. As demonstrated in the results of trend topics, the “deep learning” has emerged as the most prominent keyword in recent years, indicating its establishment as a key tool in cDMRI research. Since low SNR and prolonged acquisition time remain major limitations of cDTI, the bibliometric findings suggest that incooperating deep learning-based reconstruction and denoising methods is an emerging research area with increasing scientific interest. The need for multiple diffusion encoding measurements to robustly reconstruct the self-diffusion tensor leads to prolonged acquisition times, while signal loss from T2 decay and diffusion attenuation results in a lower SNR (41). Deep learning could reduce data requirements in STEAM acquisition maintaining similar image quality (42). Phipps et al. developed and assessed a denoising convolutional neural network based on residual deep learning, achieving at least a twofold acceleration for in-vivo cDTI (42). Phipps et al. quantified the SNR, structural similarity index, MD, FA, and helix angle transmurality, and found no significant differences (42). To address motion-induced signal loss, Zhu et al. proposed an unsupervised dense-encoder-fusion-decoder network for cDWI (43). The cooperation of these networks could investigate the *in-vivo* myocardial structure more accurately. In addition, utilizing deep learning to reconstruct cDTI index maps from just six raw cDWI images could achieve lower error rates than traditional methods. Liu et al. developed a deep learning framework, FG-Net, to obtain high-quality cDTI parameter maps from six cDWI images by combining deep-learning-based image generation with tensor fitting. They indicated that FG-Net could generate inter-directional cDWIs from six input cDWIs to supplement the missing information and improve the estimation accuracy of cDTI parameters (44). Ferreira et al. found that diffusion tensor prediction using a trained networks is a promising approach for reducing the number of breath-holds required in cDTI studies. Their proposed U-Net method reduced the cDTI acquisition from approximately 12 breath-holds to just 3 (31).

Though deep learning could be used a novel tool for cDMRI, the bias in reconstructing diffusion tensor maps inevitably exist. Whether this bias caused by deep learning-based methods were acceptable remained unknown. In addition, the evaluation and validation of the deep-learning based methods in cDMRI have not been established yet. Recent studies have recognized the cDTI indices derived from the images acquired from non-deep-learning-based method as the reference standards. Though it is reasonable, however, the accuracy and reproducibility of cDTI are still needed to be confirmed so far. The cDTI indices derived from deep-learning based methods are encouraged to be validated against histology, similar with Nielles-Vallespin et al. in 2017. Moreover, the generalization ability of deep learning-based methods should be validated using external test sets in recent studies. However, no cDMRI studies employing deep learning methods have utilized external test sets to date. This may be attributed to the lack of standardization and interpretability of cDMRI protocols, which severely limits the inclusion of external test set cohorts. Hence, there are several recommendations provided to the cDMRI researchers: First, the cDTI indices derived from deep-learning based methods are encouraged to be validated against histology if possible. Second, these methods are also encouraged to be validated in a large-scale, multi-center, multi-vendor studies.

### 4.4 Limitations

Several limitations of the present study should be acknowledged. This study only included English-language publications indexed in the WoSCC and PubMed, which, although widely used for bibliometric analysis, may exclude relevant research from other databases (e.g., Scopus or Embase) and non-English sources. Additionally, the search period from 2005 to 2024 means that the most recent articles may have been missed. Citation based analyses inherently favour older publications, whereas recently published high-quality studies may not yet have accumulated sufficient citations. The self-citations were included in the citation analyses, which led to self-citation effects. Citation bias may also exist, as self-citations were included in the citation analysis and a small number of highly cited landmark studies may exert disproportionate influence on the identified research hotspots and trends. The keyword bursts and trend topic analyses should not be considered as direct predictors of future cDMRI's development. Other limitations include the inability of bibliometric analyses to assess methodological quality and the disproportionate influence of highly cited landmark publications. Despite these limitations, our comprehensive dataset provides an up-to-date, systematic overview of the research landscape in cDMRI, offering valuable insights to guide future investigations.

## 5 Conclusion

To our knowledge, this is the first dedicated bibliometric analysis focusing on cDMRI research. The present study primarily analyzed the research hotspots and future trends in cDMRI. The “diffusion tensor imaging” ranked as the most prominent keyword with the highest occurrence count and strongest citation bursts. The top-cited articles with the strongest citation bursts focused on three main areas, including clinical application of cDTI, sequence design of cDMRI, and cardiac electrophysiology based on cDTI. Trend topic analysis further identified “deep learning” as an emerging research focus in cDMRI since 2022.

## Data Availability

The original contributions presented in the study are included in the article/Supplementary Material, further inquiries can be directed to the corresponding author/s.
