# Supplementary material for: Future of cardiac diffusion magnetic resonance imaging: a bibliometric analysis
Source: Front Med Technol. 2026 Oct 13;8:1801878. doi: 10.3389/fmedt.2026.1801878 (PMC13710368; doi:10.3389/fmedt.2026.1801878)
Supplement: Supplementary file 1 [file Datasheet1.pdf]

Supplementary Material

1 Results

Figure S1 illustrates the international collaboration network in cDMRI research. Table S1 ranked the top 10 most active countries, with The United States leading in both publication count (157 articles) and total citations (6313). The United Kingdom follows with 124 articles and 3170 citations, while China ranks third with 69 articles. Notably, the United Kingdom had the highest centrality (0.55) and total link strength (120), slightly exceeding the United States (centrality 0.52, total link strength 105), indicating its pivotal role in global collaboration. FigureS2 presented the institutional collaboration network.

Table S2 identified the top authors in cDMRI research. Teh Irvin led with 27 publications, followed by Kozerke Sebastian (26 articles) and Stoeck Christian T (24 articles). In terms of citation impact, Nielles-vallespin Sonia ranked first (838 citations), followed by Pennell Dudley J (808 citations) and Kozerke Sebastian (766 citations). Figure S3 visualized the co-authorship network, while Figure S4 maps the relationships among between the most frequently cited authors.

2 Supplementary Figures and Tables

2.1 Supplementary Figures

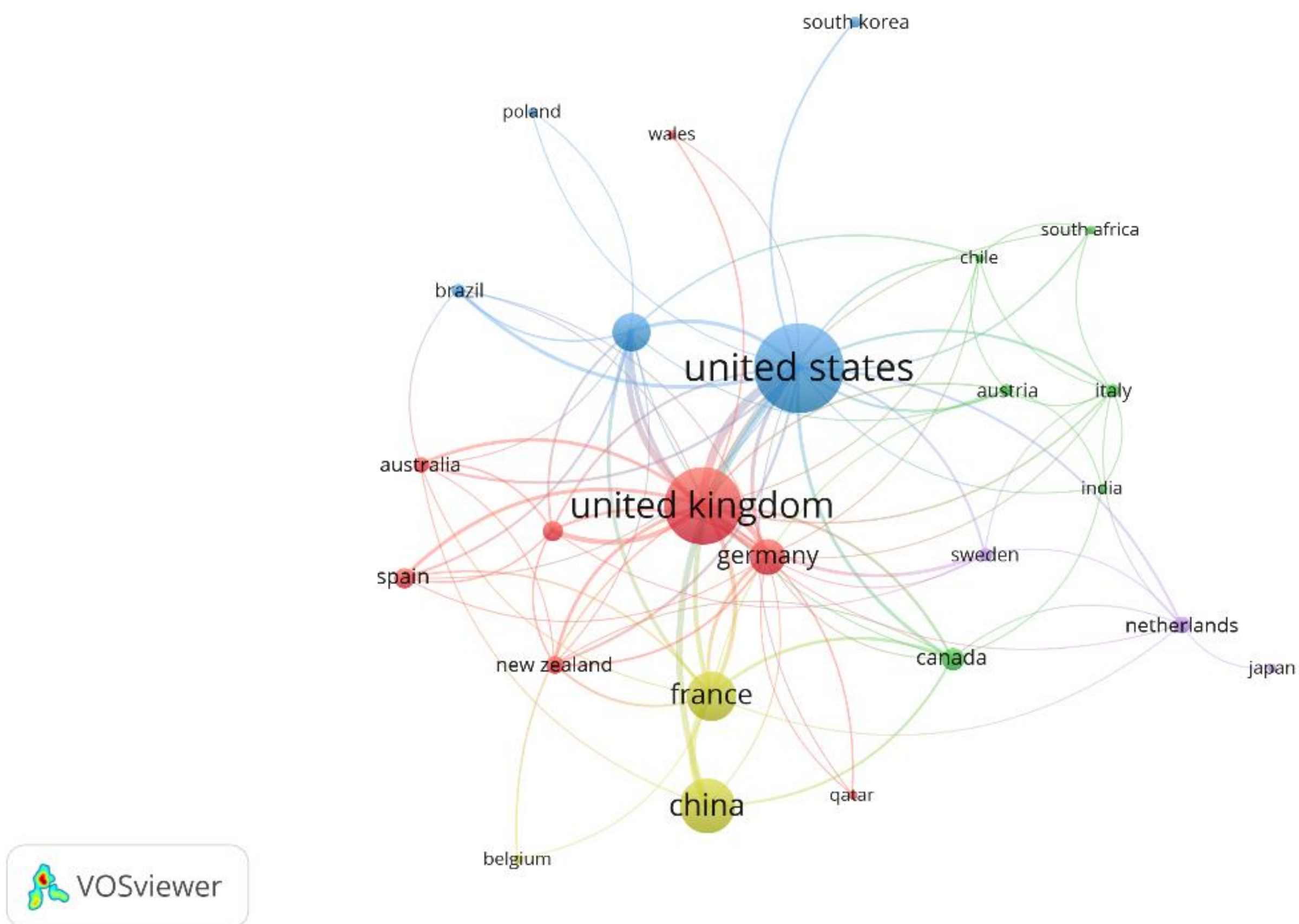

Revised Figure S1. Collaboration network analysis of authors' countries.

The width of links between nodes represented the strength of association between author countries, with thicker links indicating stronger relationships. The size of each node corresponded to the occurrence frequency of author countries, where larger node size denoted the author countries that appeared more frequently. Nodes sharing the same color belonged to the same cluster.

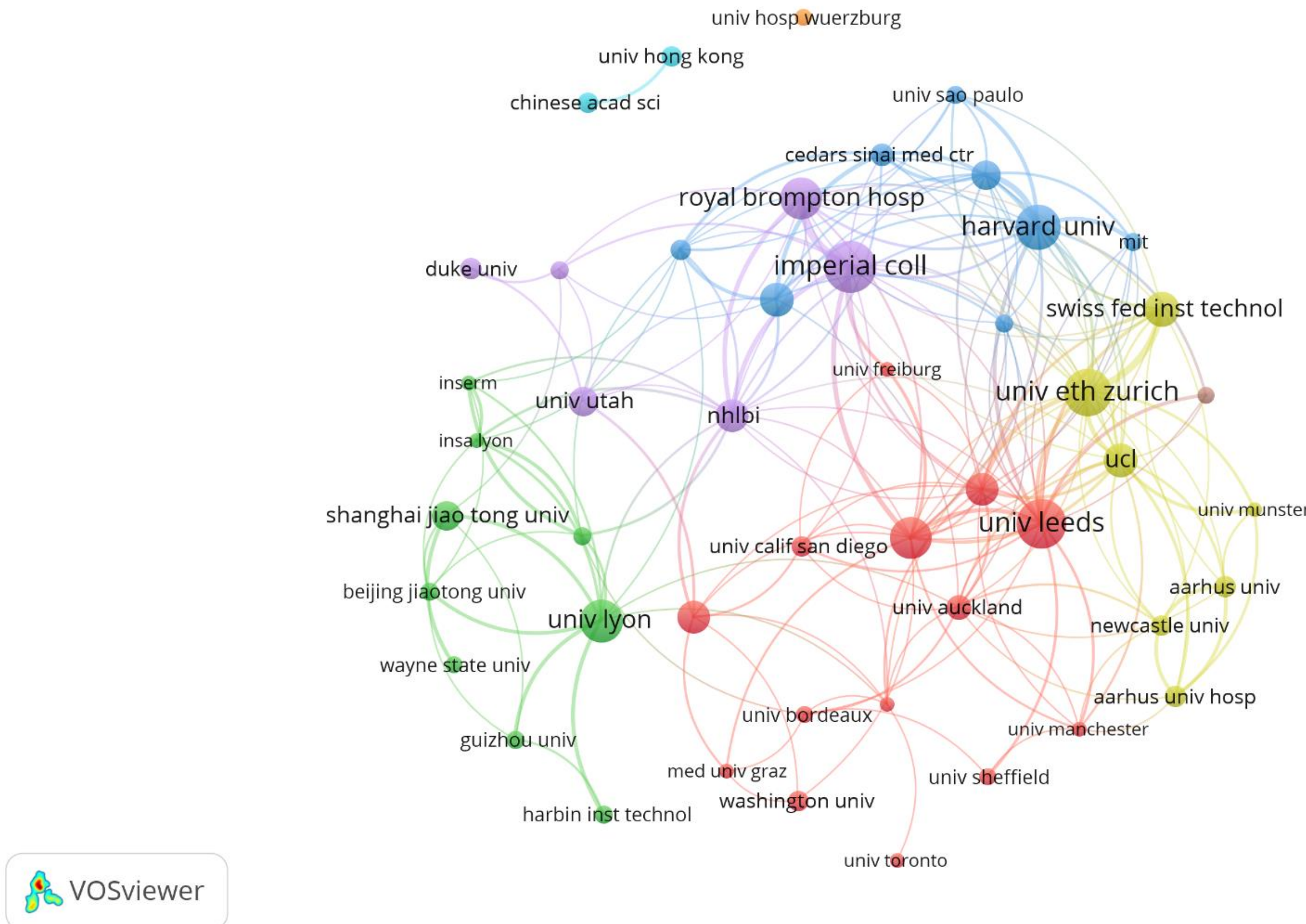

Revised Figure S2. Collaboration network analysis of institution.

The width of links between nodes represented the strength of association between authors' institution, with thicker links indicating stronger relationships. The size of each node corresponded to the occurrence frequency of institution, where larger node size denoted institution that appeared more frequently. Nodes sharing the same color belonged to the same cluster.

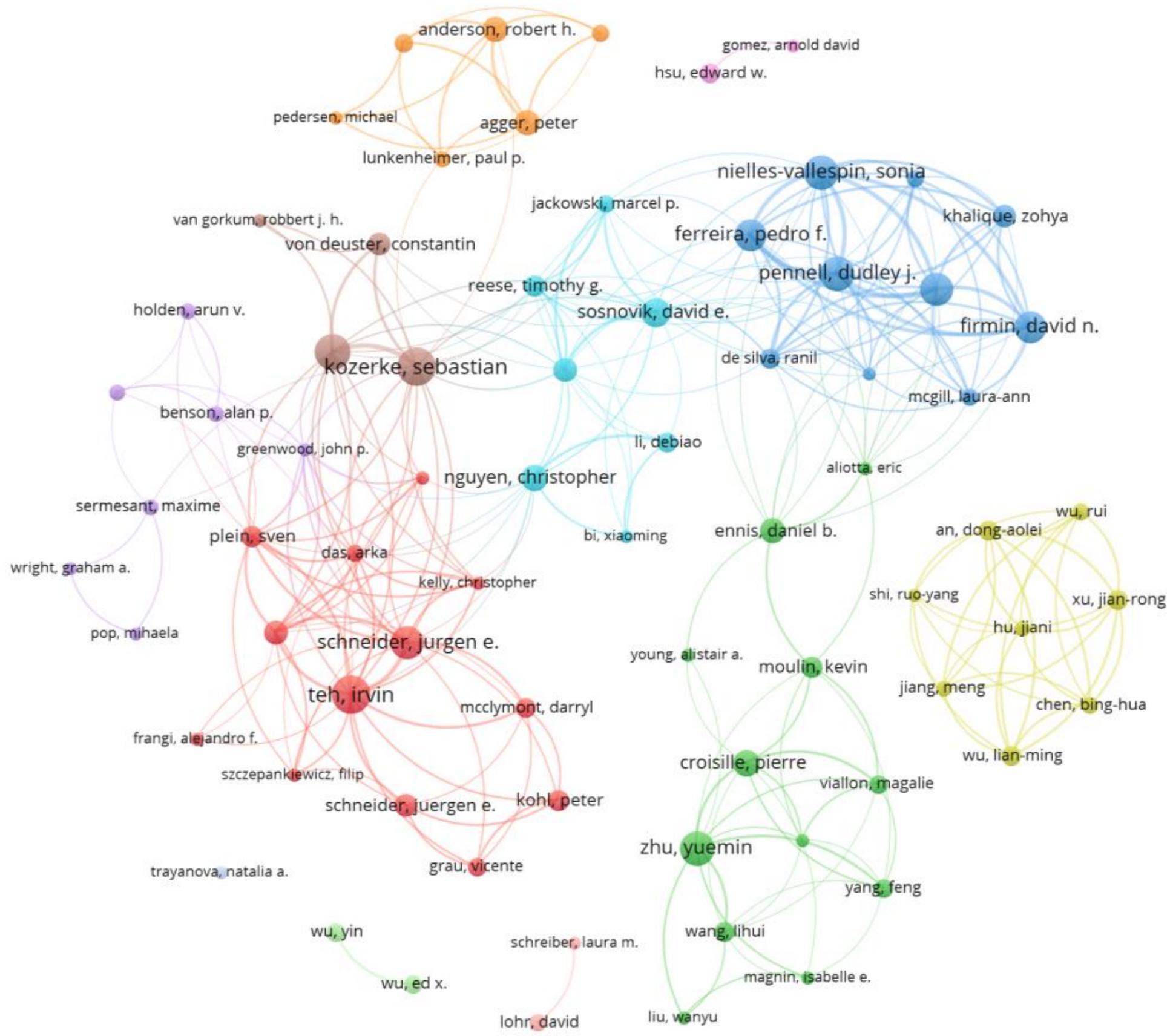

Figure S3. Collaboration network analysis of authors.

The width of links between nodes represented the strength of association between authors, with thicker links indicating stronger relationships. The size of each node corresponded to the occurrence frequency of authors, where larger node size denoted institution that appeared more frequently. Nodes sharing the same color belonged to the same cluster.

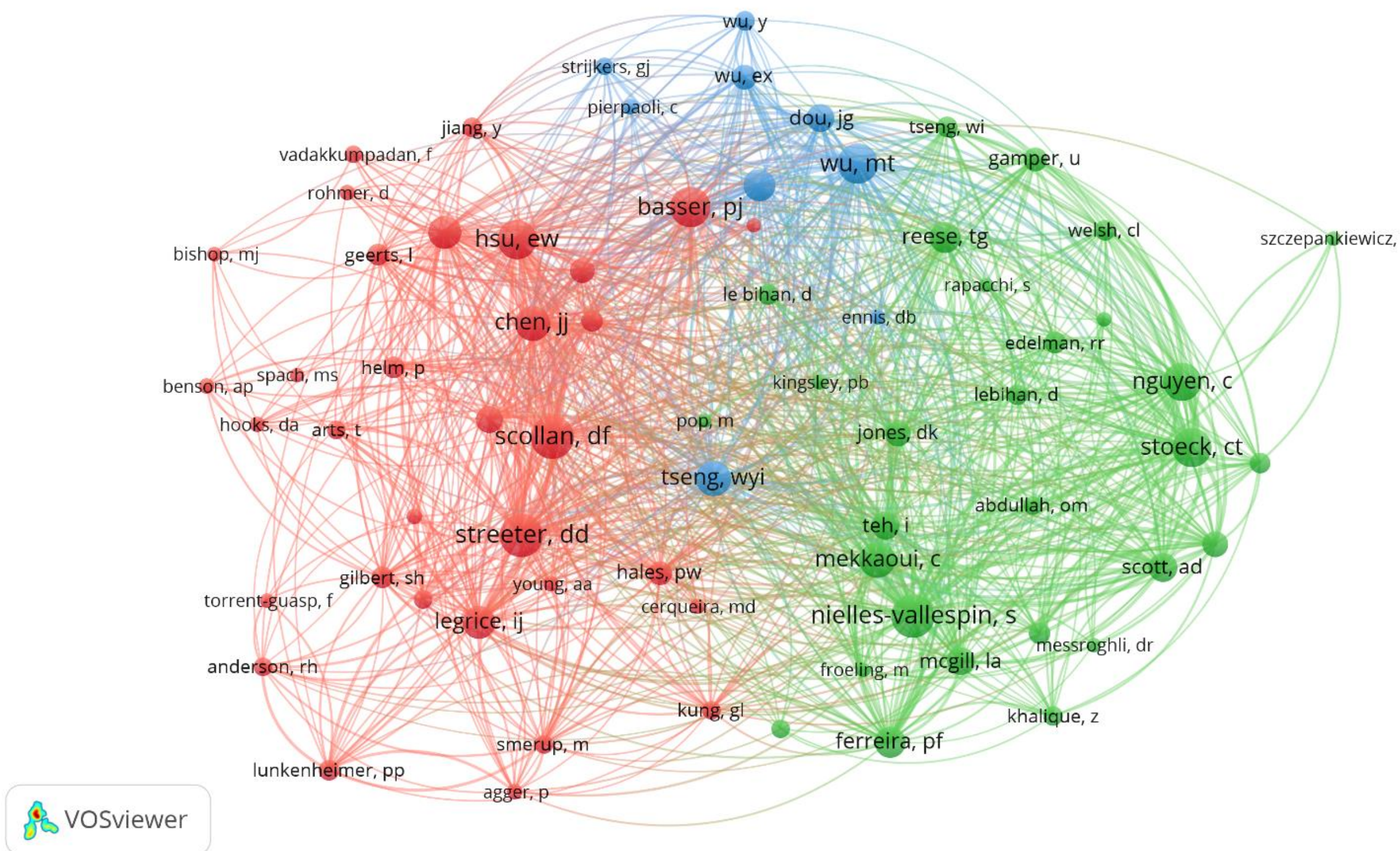

Figure S4. Co-citations network analysis of authors.

The width of links between nodes represented the strength of association between author citations, with thicker links indicating stronger relationships. The size of each node corresponded to the occurrence frequency of author citations, where larger node size denoted institution that appeared more frequently. Nodes sharing the same color belonged to the same cluster.

Table S1. The top 10 productive countires/regions in cardiac diffusion magnetic resonance imaging research

Country	Documents	Centrality	Citations	Total link strength
United States	157	0.52	6313	105
United Kingdom	124	0.55	3170	120
China	69	0	1112	47
France	58	0.09	1237	68
Switzerland	39	0.15	1026	52
Germany	34	0.19	840	51
Canada	16	0.04	529	22
Denmark	14	0.02	271	27
Spain	13	0.02	206	16
New Zealand	11	0.01	425	20

Table S2. Top 25 authors in cardiac diffusion magnetic resonance imaging research

Author	Documents	Citations	Total link strength
Teh, Irvin	27	358	61
Kozerke, Sebastian	26	766	59
Stoeck, Christian t.	24	641	58
Nielles-vallespin, Sonia	23	838	94
Zhu, Yuemin	23	215	9
Pennell, Dudley J.	22	808	91
Schneider, Jurgen E.	21	278	49
Scott, Andrew D.	21	686	88
Ferreira, Pedro F.	19	664	83
Firmin, David N.	19	696	79
Sosnovik, David E.	17	720	37
Croisille, Pierre	14	488	12
Nguyen, Christopher	14	365	12
Agger, Peter	13	268	11
Anderson, Robert H.	13	303	12
Ennis, Daniel B.	13	419	10
Dall'armellina, Erica	12	98	41
Mekkaoui, Choukri	12	534	33

Khalique, Zohya	11	335	45
Schneider, Juergen e.	11	289	16
Von deuster, Constantin	11	385	24
Kohl, Peter	10	394	14
Moulin, Kevin	10	157	10
Plein, Sven	10	132	33
Reese, Timothy G.	10	679	23

Table S3. Thesaurus mapping table

Words	Thesaurus
China	Peoples's R China, Taiwan
diffusion tensor imaging	cardiac diffusion tensor imaging, cardiac dti, dti, diffusion tensor mri, diffusion tensor imaging (dti), diffusion magnetic resonance imaging, diffusion tensor, cardiac diffusion tensor imaging (cdti), cardiac diffusion tensor imaging (dti), cardiac diffusion tensor mri, diffusion tensor cardiac magnetic resonance imaging, diffusion tensor cmr, diffusion tensor magnetic resonance imaging, diffusion tensor magnetic resonance imaging (dt-mri), dt-cmr, cardiac diffusion tensor, magnetic resonance diffusion tensor imaging, mr-diffusion tensor imaging, tensor mri, in vivo cdti, dt mri
cardiac	heart, rat heart, heart structure, human cardiac, human cardiac, in vivo human heart
magnetic resonance imaging	cardiovascular magnetic resonance, mri, cardiac mri, cardiac magnetic resonance, cardiovascular magnetic resonance imaging, cardiac magnetic resonance, cmr,

	cardiac magnetic resonance (cmr) imaging, cardiac magnetic resonance imaging, cardiovascular mri, magnetic resonance, functional cmr
diffusion weighted imaging	cardiac diffusion-weighted imaging, diffusion-weighted imaging, dwi, cardiac diffusion weighted imaging, diffusion-weighted cardiac mri (dwi), diffusion-weighted images, diffusion-weighting, diffusion mri, diffusion weighted mri, diffusion weighting, diffusion weighted mr
diffusion	cardiac diffusion, cardiac diffusion mri, cardiac diffusion imaging, diffusion cardiovascular magnetic resonance, diffusion imaging, water diffusion
intravoxel incoherent motion	ivim
cardiac microstructure	microstructure, 3d cardiac microstructure, myocardial microstructure
myocardium	myocardial architecture, myocardial structure, left ventricular myocardium
deep learning	cnn, convolutional neural network

spin echo	spin echoes
perfusion	perfusion mri, microvascular perfusion
motion effect	motion effects
model	models
mathematical model	mathematical models
late gadolinium enhancement	late-gadolinium enhancement, late gadolinium enhanced imaging
laminar structure	laminar sheets, laminar architecture
echo planar imaging	echo planar images, echo planar
compressed sensing	compressed sense
cardiac fiber	fiber architecture, fiber, fiber structure, fiber orientation, cardiac myofiber arrangement, cardiac fibers, cardiac fiber structure, cardiac fiber orientation, cardiac fiber architecture
myocardial infarction	infarction, chronic myocardial infarction, chronic infarction, acute myocardial infarction

motion compensation	motion compensation and analysis, higher-order motion compensation, compensation
UNITED STATES	USA
United Kingdom	England, Ireland, Scotland
